# Tourette Syndrome and Obsessive-Compulsive Disorder: Event-Related Brain Potentials Show Similar Mechansims of Frontal Inhibition but Dissimilar Target Evaluation Processes

**DOI:** 10.1155/2003/326468

**Published:** 2003-01-01

**Authors:** Sönke Johannes, Bernardina M. Wieringa, Wido Nager, Dominik Rada, Kirsten R. Müller-Vahl, Hinderk M. Emrich, Reinhard Dengler, Thomas F. Münte, Detlef Dietrich

**Affiliations:** ^1^Department of NeuropsychologyOtto-von-Guericke UniversitätMagdeburgGermany; ^2^Department of NeurologyMedizinische Hochschule HannoverGermany; ^3^Department of Clinical Psychiatry and PsychotherapyMedizinische Hochschule HannoverGermany

**Keywords:** tourette syndrome, obsessive-compulsive disorder, event-related potentials

## Abstract

*Objectives:* Tourette Syndrome (TS) and Obsessive-Compulsive Disorders (OCD) share many clinical similarities and show a strong comorbidity. Current theories view a frontal-striatal dysfunction as the underlying cause of many clinical aspects of both disorders.

This study sought to investigate mechanisms of conceptual integration and attention in both disorders. We hypothesized that the processing of stimuli with interfering aspects would be altered in a similar way while attentional mechanisms could differ.

*Methods:* Event-related brain potentials (ERPs) were recorded in a modified STROOP-paradigm in groups of TS and OCD patients and in a control group. The paradigm involved the presentation of color words in a range of different colors. The subjects had to respond to words of matching word content and color and to ignore mismatching stimuli.

*Results:* Incongruent stimuli elicited a frontal negative component (“N450”) which was enhanced in amplitude and prolonged in latency in both patient groups. Matching stimuli evoked enhanced N2 and P3b components representing target evaluation mechanisms. The OCD group alone displayed a larger P3b amplitude in comparison to both other groups.

*Conclusions:* The data are interpreted to indicate that frontal inhibitory mechanisms are altered alike in TS and OCD. In contrast, only the OCD group showed evidence for aberrant target evaluation.

## 1. Introduction

The Tourette Syndrome (TS) is a complex neuropsychiatric disorder which can be diagnosed in the presence of multiple fluctuating motor and vocal tics after the exclusion of other diseases [42]. In his first description of the syndrome, Tourette himself had included Obsessive-Compulsive thinking as part of the disease spectrum [23]. The diagnosis of Obsessive-Compulsive Disorder (OCD) can be made in the presence of obsessions and compulsions that cause marked distress [2].

There is a strong comorbidity between TS and OCD.

ISSN 0953-4180/03/$8.00 © 2003 – IOS Press. All rights reserved

A number of studies have shown that 40% to 65% of TS patients do also fulfill the diagnostic criteria for OCD and have established links to genetic factors of the diseases [16]. In fact, data from these studies suggest that both, TS and OCD, can be viewed as different manifestations of the same underlying disorder. This is, of course, not equivalent with the notion that all individuals with OCD share a common etiology.

Alterations of basal ganglia and forebrain have been described to occur in TS as well as in OCD. These include changes of neuroanatomy [39], blood flow [26], metabolism [7], functional activation [5], electrophysiology [38], cortical excitability [15,45] and neurochemistry [14,28], A number of similarities between the findings for both disorders have given rise to the hypothesis that a frontal-striatal dysfunction is underlying many clinical aspects of TS and OCD. This hypothesis is supported by a number of neuropsychological studies which have documented impaired frontal executive functions in both disorders (e.g. [6,17,34]).

The findings in other neuropsychological domains, however, remain heterogeneous. A critical review of the data suggests that both disorders are associated with only minor deficits if examined with conventional methods (reviewed in [1]).

In this situation event-related brain potentials (ERPs) have proven to be an important tool to elucidate neuropsychological deficits in both disorders. ERPs are small voltage fluctuations which can be recorded non-invasively from the intact human scalp. While some early components vary as a function of physical stimulus parameters, other components of longer latencies only appear in conjunction with specific perceptual or cognitive processes.

With the use of ERPs it has been possible to identify changes of attentional mechanisms in TS [12,36,43]. In a detailed analysis Johannes et al. [22] documented that Tourette patients do consistently show an enhanced effort in a number of attentional experiments of different complexity. Their performance is normal in easy but reduced in complex tasks. The findings for OCD patients, in contrast, differ from this profile. A number of studies have shown that several attentional mechanisms are changed [10,11,27]. A direct comparison between both disorders is needed, however, to allow a firm statement as to their differences in the attentional domain.

The aim of this study was provide such a comparison. ERPs were recorded in a modified STROOP paradigm in groups of OCD and TS patients and in a control group. The paradigm involved the presentation of color words in matching (e.g., the word "red" displayed in red color) and mismatching colors (e.g., the word "red" displayed in green color). The subjects' task was to respond to all matches. This necessitates the conceptual integration of word content and color information. Mismatches cause a reliable and stable interference effect which is regarded as one measure of executive functions [41]. Matches and Mismatches elicit a number of ERP components [25]. Recently West and Alain [44] have confirmed that a frontally located negativity (termed N450) reflects the activity of a neural system involved in the suppression of a conceptual level processing system on incongruent trials. We constructed our experimental task such that incongruent trials would elicit a large N450 component. Additionally our goal was to analyze attentional processes within the same experiment. Therefore we chose an experimental setting which required the subjects to respond to the appearance of infrequently occurring congruent stimuli. Under such conditions congruent stimuli elicit large centro-parietal negativities (N2 component) and parieto-temoral positivities (P3b component). These components have been widely studied and do functionally represent meaning-based conceptual level evaluation and identification processes (e.g. [18,33]).

We hypothesized that both, TS and OCD patients would show altered frontal N450 ERP components compared to the control group reflecting altered mechanisms of conceptual integration. In contrast, the target identification process was easy to perform so that we expected the TS group to exibit unchanged N2 and P3b components. The predictions for the OCD group were less precise because the data about attentional processes in OCD are heterogeneous. We deemed it possible that the chosen paradigm would reveal an altered P3b component in the OCD group.

## 2. Patients and methods

Thirteen native German speaking adults were diagnosed to fulfill the DSM IV criteria for the diagnosis of Tourette syndrome [2]. The data of two of them had to be excluded from analysis because they were contaminated with artifacts from excessive eye movements and blinking. Due to a technical error the data of an additional subject were lost. The characteristics of the remaining 10 TS patients are shown in Table 1. None was free of symptoms. Two patients fulfilled the DSM IV criteria for OCD, two other patients the diagnosis of Attention Deficit Hyperactivity Disorder (ADHD) and

Table 1
Group characteristics

| **TS (CS)** Patient no. | Sex | Age (years) | Educ. (y) | H | OCS |
|---|---|---|---|---|---|
| 1 | m (m) | 64 (59) | 13 (13) | r | 1 |
| 2 | m (m) | 51 (49) | 13 (13) | r | 2 |
| 3 | m (m) | 25 (26) | 13 (13) | r | 3 |
| 4 | m (m) | 24 (23) | 13 (13) | r | 3 |
| 5 | m (m) | 43 (45) | 8 (8) | r | 1 |
| 6 | m (m) | 38 (34) | 9 (10) | r | 2 |
| 7 | f (f) | 20 (22) | 13 (13) | r | 2 |
| 8 | m (m) | 16* (16)* | 10* (9)* | r | 1 |
| 9 | m (m) | 39 (37) | 13 (13) | r | 1 |
| 10 | m (m) | 24 (26) | 12 (12) | r | 3 |
| mean+− standard deviation | | 34.4+−15,3 (33.7)+−13,7 | 11.7 (11.7) | | |
| **OCD** | | | | | |
| 1 | f | 45 | 10 | r | cleaning |
| 2 | f | 30 | 13 | r | checking |
| 3 | f | 22 | 13 | r | checking |
| 4 | f | 35 | 10 | r | washing |
| 5 | f | 25 | 13 | r | cleaning |
| 6 | f | 29 | 10 | r | thinking |
| 7 | f | 46 | 10 | r | checking |
| 8 | m | 40 | 13 | r | checking |
| 9 | m | 29 | 10 | r | cleaning |
| 10 | f | 49 | 10 | r | washing |
| mean+− standard deviation | | 35.0+−12,8 | 11.2 | | |

*Refers to subjects who were still in education.
Educ.: education.
TS: Tourette Syndrome group.
CS: Control group.
OCD: Obsessive-Compulsive Disorder group.
H: handedness.
OCS: Obsessive-Compulsive symptoms.
ADHD: attention deficit hyperactivity disorder.
NL: Neuroleptics.
It can be seen that TS, OCD and control group share similar characteristics.

an additional subject the criteria for both disorders. Ten neurologically healthy control subjects were recruited from the hospital staff/students (4) and from patients (6) hospitalized for peripheral trauma. Careful examination by a neurologist (SJ) ruled out head trauma and trauma to a body-part impeding performance of the neuropsychological tasks (e.g. fracture of the dominant upper extremity, neck trauma). Similarly, a history of neurological or psychiatric disease and current intake of centrally acting drugs were exclusion criteria for control subjects. Controls and patients were matched with respect to age (within 5 years), sex, handedness (as assessed by the Edinburgh Handedness Inventory [30]) and education (see Table 1). A pairwise match was attempted.

Ten OCD patients were selected from a larger study population. Care was taken that these OCD subjects matched the TS patients as best as possible. However, it was impossible to resolve certain gender differences between groups. A good match was obtained with respect to education and handedness. Although the age ranges differed between groups this effect was not significant. Pharmacological treatment and the existence of depression or phobias were exclusion criteria for these patients (details in Table 1).

All participants gave their informed consent in written form.

### 2.1. *Stimulus material*

The words “red”, “blue”, “green”, and “yellow” were shown slightly above a fixation mark located at the center of the dark gray background of a video monitor. The colors red, blue, green and yellow were used to present the words. All 16 possible combinations between word content and presentation color were used.

This resulted in four stimuli with matching content and color (e.g., the word “blue” presented in blue color) and 12 mismatches (e.g., the word “blue” presented in green color). Each content/color combination was used 30 times. Thus, there was a total of 120 “matches” and 360 “mismatches”. The stimuli were shown one at a time for 300 ms in a pseudorandom order and the interstimulus interval varied randomly between 1200 ms and 1900 ms (rectangular distribution). The words subtended 2–3° of visual angle in horizontal and 0.6° in vertical diameter.

### 2.2. Procedure

Prior to and during the experiment, the subjects were required to maintain eye fixation on a central fixation point. The subjects were instructed to press a button held in the right hand as quickly and as accurately as possible whenever a “match” appeared.

The responses were classified as hits when they occurred within a timeperiod from 200 ms to 1200 ms after the presentation of a “match”.

### 2.3. Recording

The electroencephalogram was recorded using tin electrodes mounted in an electrode cap (Electro-Cap International) from FP1, FP2, F3, F4, F7, F8, C3, C4, FZ, CZ, PZ, T3, T4, T5, T6, P3, P4, O1, O2, of the international 10–20 System [20]. Electrode impedances were maintained below 10 kΩ. The time constant was 10 seconds and the low pass filter 100 Hz. AD conversion was done with a sample rate of 256 Hz. Ocular fixation was verified by recordings of the horizontal electrooculogram (EOG). Eye blinks were detected with the vertical electroencephalogram. All scalp electrodes were referenced to an electrode located on the right mastoid and rereferenced off-line to a balanced non-cephalic sterno-vertebral channel [37].

Behavioral performance was analyzed in terms of reaction time and hit rate.

### 2.4. Quantification

ERPs were averaged off-line in a timewindow of 1024 ms beginning 100 ms before stimulus onset. Trials contaminated with ocular or muscle artifacts were excluded from the analysis. The inspection of the control subjects’ waveforms ensured the existence of the relevant frontal N450 and central N2 and parietal P3b ERP components. The components were present in all individual control subjects.

Difference waves were calculated by subtracting the ERPs to matches from the ERPs to mismatches in case of N450 and by subtractions in reverse order (matches minus mismatches) in case of N2 and P3b. ERPs were classified in terms of mean amplitudes of the difference waves over the scalp sites with maximal activity during indicated time periods and in terms of the components’ peak latencies. The time periods were centered around the ERP components’ peaks of the grand-average waveforms which resulted from collapsing all the individual subjects’ waveforms together.

Scalpsites and timewindows used for analysis were:

frontal N450: FP1/2 electrodes, 200–500 ms post stimulus central N2: C3/4 electrodes, 300–600 ms post stimulus parietal P3b: PZ electrode, 500–800 ms post stimulus

These electrode sites were chosen because they showed the maximal ERP effects in the control group. A subsequent analysis included the additional electrode sites CZ and P3/4. Because this did not result in a different statistical outcome of the analysis the findings are not reported here.

Repeated-measures analyses of variance (ANOVA) were performed on behavioral and ERP data. The factor group (TS patients vs. OCD patients vs. control group) served as between-groups variable and Bonferroni tests were performed to compare each two groups. The analysis of the frontal N450 and central N2 components included the within-groups factor hemisphere of recording (left vs. right). This analysis revealed a significant interaction between the factors group and hemisphere of recording for the frontal N450. Therefore a subsequent analysis of the N450 was done separately for the left and right hemispheres. All analyses were adjusted for nonsphericity with the Greenhouse-Geisser epsilon coefficient [21].

## 3. Results

Figure 1 shows the control group’s grand average ERPs to stimuli with matching and mismatching features. At the frontal sites FP1 and FP2, between 200 and 500 ms post stimulus, mismatches elicit a more negative waveform than matches. This is the frontal N450 component. At the central sites C3 and C4 a negative waveform peaking between 300 and 600 ms

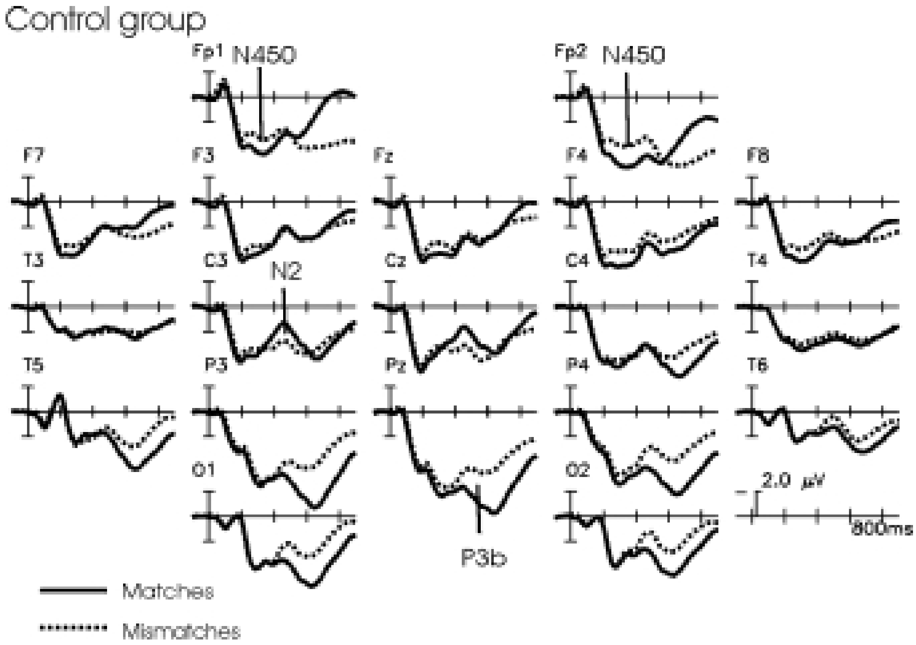

Fig. 1. Grand average waveforms of the control group. Stimuli of dissimilar content and color elicit a more negative waveform (denoted as "N450") than stimuli with "similar" features over the frontal electrodes FP1 and FP2 between 200 and 500 ms after presentation. Stimuli elicit a waveform with a negative peak (denoted as "N2") over the central electrodes between 300 and 600 ms after presentation and a more positive waveform (denotes as "P3b") over the parietal electrodes beteween 450 and 900 ms post stimulus.

post stimulus can be seen. This is the central N2 component. Matches elicit a more positive waveform at the parietal electrodes P3, P4 and PZ between 500 and 900 ms post stimulus, constituing the P3b component.

Figure 2 displays the N450 difference waves at the sites FP1 and FP2 for all three groups. It can be seen that the onset does not differ between groups but that the amplitudes were larger and peak latencies were delayed in the TS and OCD-patient groups compared to the control group.

Statistically, the latency differences were reflected in a significant main effect of the factor group for the peak latency measures ($F[2,27] = 3.75$; $p < 0.04$). The pairwise comparisons revealed significant differences between TS and control group and between OCD and control group but not between TS and OCD ($p < 0.05$; $p < 0.02$; $p > 0.8$, respectively).

The amplitude differences were pronounced over the left hemisphere. Statistically, this was represented by a significant interaction between the factors group and hemisphere of recording ($F[2,27] = 3.5$; $p < 0.05$) for the amplitude measures. The separate analysis for the FP1 electrode showed a significant effect for the factor group ($F[2,27] = 4.2$; $p < 0.025$) and the pairwise comparison revealed significant differences between TS and control group and between OCD and control group but not between TS and OCD ($p < 0.002$; $p < 0.03$; $p > 0.9$, respectively).

Figure 3 displays the central N2 difference waves at the scalpsites C3 and C4. It can be seen that the OCD patients' waveform is more negative than the other waveforms, particularly at C4. Statistically, this was reflected by a significant effect of the factor group ($F[2,27] = 3.4$; $p < 0.05$). The pairwise comparison revealed significant differences between OCD and control group but not between OCD and TS or between TS and control group ($p < 0.03$; $p > 0.1$; $p > 0.5$, respectively). The insignificance of the differences between OCD and TS group may have been caused by the fact that the TS group showed a strong negativity at the site C3 but not at the site C4. Although this hemispheric asymmetry may be related to the pathophysiology of the underlying disorders an in-depth analysis of this effect was beyond the scope of the present publication. There were no significant latency differences between groups.

Figure 4 displays the central P3b difference waves at the scalpsite PZ. It can be seen that the OCD patients' waveform is of greater amplitude than the other waveforms. Statistically, this was reflected by a significant effect of the factor group ($F[2,27] = 4.0$; $p < 0.03$). The pairwise comparison revealed significant differ-

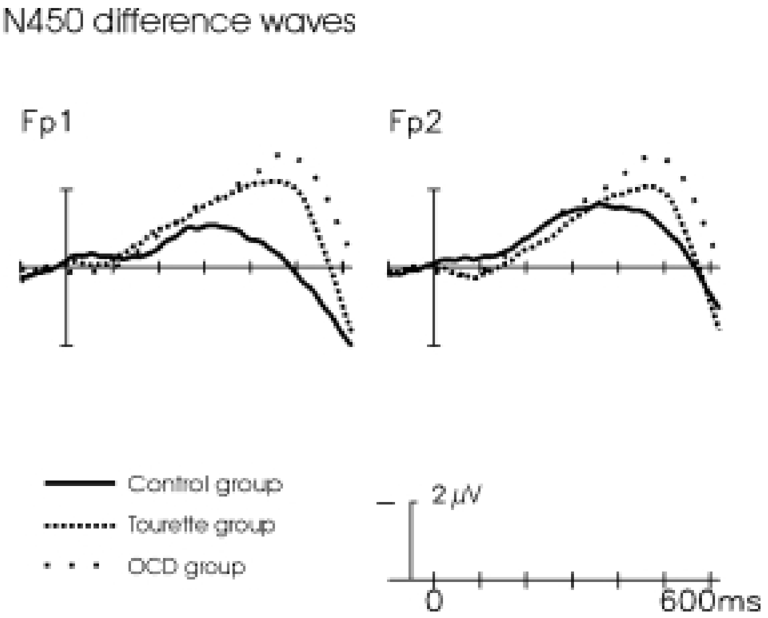

Fig. 2. “N450” difference waves over the frontal scalpsites FP1/Fp2 resulting from subtraction of the grand average waveform to “similar” stimuli from the grand average waveform to “dissimilar” stimuli. It can be seen that the TS and OCD groups display a stronger negative waveform than the control group.

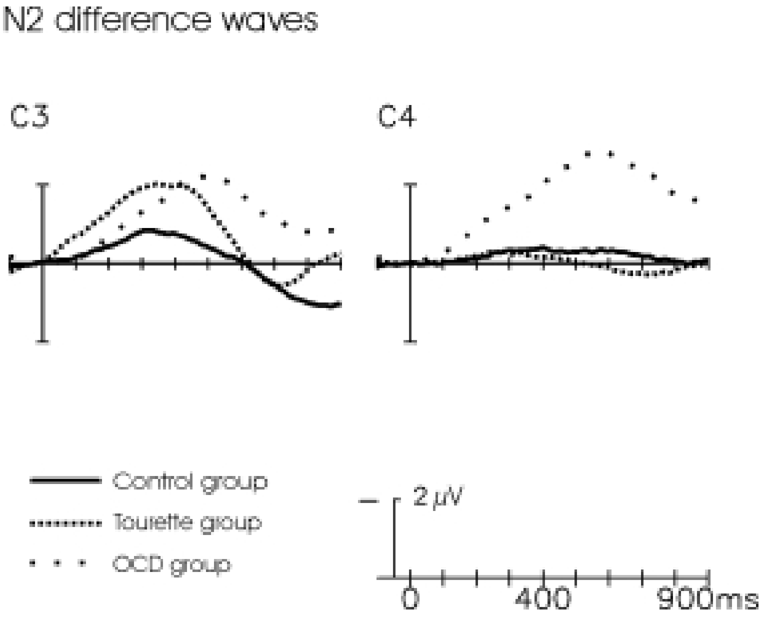

Fig. 3. “N2” difference waves over the central scalpsites C3/C4 resulting from subtraction of the grand average waveform to “dissimilar” stimuli from the grand average waveform to “similar” stimuli. It can be seen that the OCD group but not the TS group displays a stronger negative waveform than the control group.

ences between OCD and control group and between OCD and TS but not between TS and control group ($p < 0.02$; $p < 0.05$; $p > 0.6$, respectively). There were no significant latency differences between groups.

Behavioral parameters:

Hit rates and reaction times did not differ significantly between groups (hit rates: TS $94+-4.4\%$; OCD $95 + -4.4\%$; control group $91 + -5.9\%$; $F[2, 27] =$

$1.2$; $p > 0.3$; reaction times: TS $592 + -62$ ms; OCD $614 + -56$ ms; control group $630 + -50$ ms; $F[2, 27] = 1.3$; $p > 0.3$).

## 4. Discussion

The experiment revealed that both, a group of TS patients and a group of OCD patients, showed an altered electrophysiological negativity (N450) over the frontal cortex when asked to analyze stimuli with matching and mismatching features. The larger amplitude and increased latency of the N450 indicate an enhanced and prolonged neural activity.

From the electrophysiological perspective the findings can be due to an activity modulation of the neural generator of the N450 itself or to an overlap of activity from other frontal sources which are functionally related. The N450 has been shown to correlate with the efficiency of conceptual integration processes [44]. Similar negative frontal ERP components are elicited under conditions which require the inhibition of responses (frontal N2) [3] or the occurrence of commission errors (error related negativity) [13]. Recent work indicates that the anterior cingulate gyrus, regions of the prefrontal cortex and a related network form the common underlying neural generators of this electrophysiologic activity [35]. It deems likely that these sources are also causal for the alteration of the N450 observed in the present experiment. In this respect the enhanced N450 component can be interpreted to indicate altered inhibitory mechanisms in TS and OCD. The characteristics of both disorders are similar as indexed by the N450 which was identical in both patient groups. This indicates a considerable pathophysiologic overlap. Support for this interpretation comes from a number of other studies. OCD as well as TS patients can display elevated interference effects and response inhibition in conventional tests [17]. Both patient groups also show an enhanced cortical excitability as demonstrated with transcranial magnetic stimulation (TMS) [15,45] and display a hyperactivity of circuits including the frontal cortex, thalamus and basal ganglia [32]. This suggests a relation of the present experimental findings to theories of a frontal-striatal dysfunction as underlying cause of the disorders.

The data also reveal, however, important pathophysiological differences between both disorders. Although the behavioral performance measures indicate that all experimental groups were well able to respond to stimuli with matching word content and color the N2 and P3b ERP measures indicate that group differences do exist. Many studies have documented that ERPs are a sensitive tool for the analysis of cognitive processes which allow the detection of subtle experimental effects not necessarily affecting conventional behavioral measures [22].

The parietal P3b and central N2 ERP components were altered in the OCD but not in the TS group as compared to the control group. However, hemispheric asymmetries of the N2 component were observed in the TS group and not in the control group. This effect was not analyzed further. Pairwise comparisons revealed significant differences between both patient groups for the P3b component. The P3b and N2 components represent mechanisms of conceptual integration and target detection (e.g. [18,33]). Neither latency nor amplitude are affected by the STROOP interference [19]. The results of an earlier study of attentional mechanisms in TS [22] led us to expect that the P3b would remain unchanged in the TS group. The present experiment involved an easy task of target detection and TS patients have been documented to be unimpaired when performing such tasks. The data of the present experiment confirm this prediction and strengthen the view that TS patients can perform well in easy attentional tasks.

The finding of an enhanced P3b amplitude in the OCD group marks a clear difference to the findings in the TS group. It adds to the growing evidence that OCD is associated with altered mechanisms of conceptual integration and target detection. Depending on physical stimulus characteristics and task complexity shorter latencies and increased amplitudes of the P3b component have been described to occur in OCD [11]. These findings have been interpreted to indicate a misallocation of cognitive resources and a hyperarousal of the cortex. The enhanced P3b component amplitude in the OCD group is in accordance with such an interpretation. It shows that OCD patients employ an altered target identification mechanism and indicates a clear pathophysiological difference between TS and OCD. To further investigate this group difference it will be the purpose of a future study to compare the data of TS subjects with OCD symptoms to those TS subjects without OCD symptoms.

Recent work demonstrates that the P3b is generated from a number of intracranial sources representing the cortico-limbic-thalamic network [9,24]. Hence, the function of some constituents of this P3b-network must be altered in OCD. It can be speculated that this is related to clinical aspects of the disorder. Doubt-

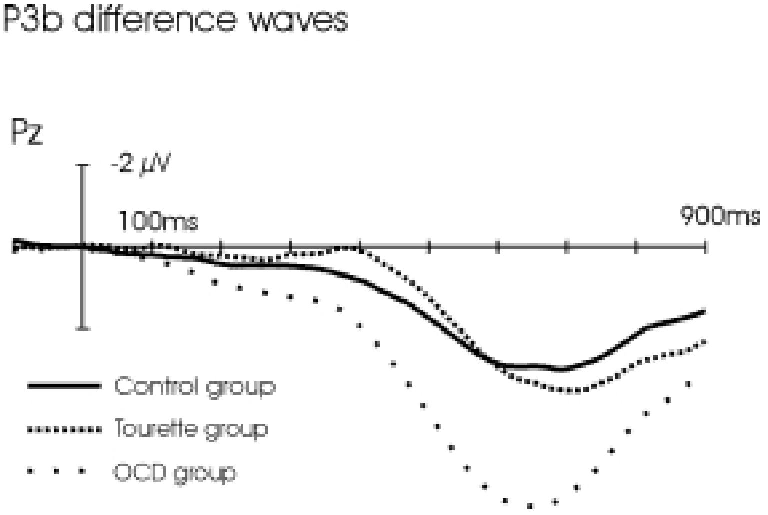

Fig. 4. "P3b" difference waves over the parietal scalpsite PZ resulting from the same subtraction as Fig. 3. The OCD displays a stronger positive waveform than the control group and the TS group.

related phenomena are a relatively unique feature of OCD [40]. Although it has long been suspected that memory deficits do play an important role in the genesis of "doubt" in OCD only minor visual and visuospatial mnestic deficits but no overall memory disturbances were documented in numerous studies within the past 100 years. The generation of the P3b, however, represents the complex interaction of heterogeneous physiological processes such as attention, attentional set shifting4 and working memory [8]. Other work has demonstrated certain P3b alterations in OCD but not in related disorders without doubt related phenomena [27]. The changed P3b amplitude of the present experiment might reflect a similar pathophysiology. It could indicate that doubt related phenomena in OCD do result from the complex interaction of a number of aberrant neuropsychological functions which are functionally embedded in the P3b-network.

One could argue that our experimental findings might in part be due to the heterogeneity of the sample groups. While all three groups were closely matched for age, education, and handedness, there were more men in the Tourette group than in the OCD group reflecting the preponderance of TS in the male gender. A critical review of the literature reveals that gender causes no reliable systematic effects on the latency or amplitude of the P3b (e.g. [29,31]). Moreover, a comparison of the P3b between female and male subjects within the OCD group revealed similar amplitudes for both genders. This analysis was of course restricted by the small number of subjects (7 female; 3 male) but supports the notion that the main experimental effects are not due to gender variations between experimental groups.

In conclusion the experimental data demonstrate that certain aspects of executive functions, i.e. inhibition, are altered alike in TS and OCD. Whereas this constitutes a strong similarity between both disorders target detection mechanisms in contrast were solely changed in the OCD group. This may be related to the clinical hallmarks of OCD.
